# Enhanced Mechanical Properties of AA6082 Matrix Composites Reinforced with B_4_C and TiO_2_: A Powder Metallurgy Approach

**DOI:** 10.1021/acsomega.5c00792

**Published:** 2025-11-24

**Authors:** Zübeyde ÖZKAN, Uğur GÖKMEN, Sema Bilge OCAK

**Affiliations:** † 37511 Gazi University, Graduate School of Natural and Applied Sciences, 06560 Ankara, Türkiye; ‡ Gazi University, Faculty of Technology, Department of Metallurgical and Materials Engineering, 06500 Ankara, Türkiye; § Gazi University, Graduate School of Natural and Applied Sciences, Department of Advanced Technologies, 06560 Ankara, Türkiye; ∥ Basic and Engineering Sciences Central Laboratory Application and Research Center, Gazi University, 06560 Ankara, Türkiye

## Abstract

This study investigates the production of B_4_C, TiO_2_, and B_4_C + TiO_2_ reinforced functional-grade composite materials (FGCM) and functional-grade hybrid composite materials (FGHCM) with an AA6082 matrix by using the powder metallurgy method at weight percentages ranging from 0 to 50%. The refractive indexes (XRD) and microstructure of the used powders were analyzed. The mechanical properties, including transverse rupture strength (TRS), and microstructural characteristics, were examined through scanning electron microscopy (SEM) and energy dispersive spectroscopy (EDS). Among the produced samples, AA6082 exhibited the highest relative density at 99.68%. The addition of reinforcement materials into the AA6082 matrix led to a decrease in the relative density of the composites. The TRS of the functionally graded materials decreased due to the nonhomogeneous distribution of reinforcement phases and the resulting notch effect. The results indicate that the reinforcement distribution should be further optimized to improve the mechanical properties, such as the transverse rupture strength of the ceramic reinforcement AA6082 composite. These findings suggest that ceramic-reinforced AA6082 composites hold potential for advanced engineering applications, although improvements in phase distribution uniformity are necessary for optimized performance.

## Graphical abstract

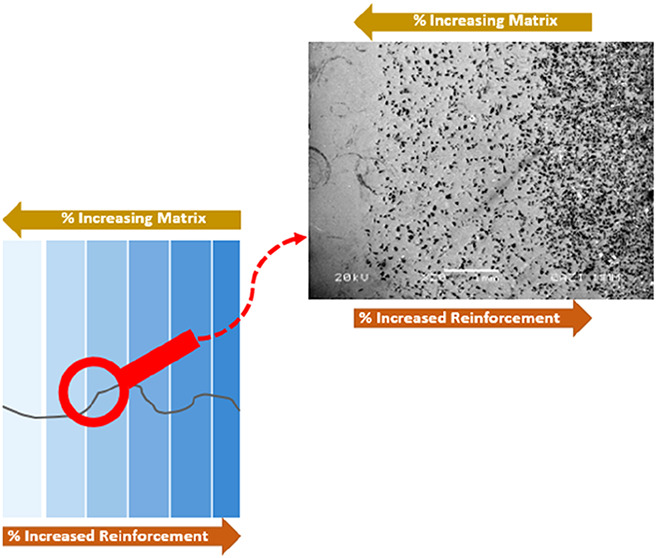

## 1 Introduction

Composite materials with nonuniform changes in composition and material structure throughout their volume are known as functionally graded composite materials (FGCM). The compositional gradient within FGCMs can be varied continuously or stepwise, depending on the design and functional requirements. FGCMs composed of ceramic and metal components can achieve the desired mechanical properties through a functionally graded design. FGCMs combine the outstanding properties of metals and ceramics in a single volume. The issues that occur during transitions between brittle and ductile materials can be mitigated by using FGMs that distribute reinforcing elements at various angles within a single structure. As a result, structures made with uniformly distributed reinforcement particles can effectively overcome this challenge.^1–3^ Due to their superior performance, FGCMs are widely applied in aerospace, bioengineering, electronic components, medicine, and ceramics.^4,5^ FGCMs can be fabricated using various methods, including electrochemical processing, laser powder bed fusion, physical vapor deposition, additive manufacturing, plasma or thermal spraying, powder metallurgy, and chemical vapor deposition.^6–8^ Among these, the powder metallurgy (PM) method effectively minimizes material losses and production costs. With PM, cost-effective machinery is used, consuming less energy while producing materials of various sizes and complex geometries at lower temperatures. Furthermore, working with powdered materials allows for the incorporation of different chemical compositions. By reduction of the pores between powder particles, this method enhances the mechanical and corrosion resistance of materials. Additionally, it is ideal for producing gradient materials, as it facilitates the gradual creation of functionally graded structures.^9–11^

Aluminum alloys, particularly AA6082, play a crucial role in creating lightweight materials for various industries, including automotive, weapon manufacturing, aerospace, electronics, and naval sectors. The low density, corrosion resistance, impressive specific strength, and outstanding thermal and electrical conductivity of these alloys make them highly valued.^12–14^ AA6082, a precipitation-hardened aluminum alloy in the Al 6xxx series, is widely used in the aerospace and automotive industries for lightweight technology due to its high specific strength, excellent machinability, and excellent thermoplastic forming ability.^15–17^ The addition of ceramic particles such as SiC, WC, TiB_2_, TiN, B_4_C, ZrO_2_, Al_2_O_3_, and TiC to aluminum alloys can significantly enhance their mechanical properties.^18–20^ TiO_2_ composites are widely used as wear-resistant coatings in industries such as machinery, printing, and textiles due to their high hardness, excellent corrosion resistance, and resistance to wear, chemicals, and heat. TiO_2_ also exhibits photoactivity, affordability, stability, and biocompatibility, making it highly beneficial for various applications, including pollution oxidation.^21,22^ B_4_C is a highly preferred ceramic material because of its exceptional hardness, chemical stability, high melting temperature, high wettability, strong bonding with alloys, high abrasive capacity, and low coefficient of thermal expansion.^23–26^

Previous studies have shown that adding 3% SiC and 0.5% TiO_2_ to Al6082 significantly improves its mechanical properties.^27^ Chao et al.^28^ demonstrated that high-strength functionally graded B_4_C/Al composites from this research could guide interface structure design and heat treatment processes. Karakoç et al.^29^ found that the Al6061 alloy had the lowest hardness, while the 12% B_4_C-reinforced composite material had the highest hardness. Their study also revealed that the 9% B_4_C + 3% SiC-reinforced composite material exhibited the highest wear resistance, owing to the good bonding of SiC to the matrix and the high hardness of B_4_C. Furthermore, Karakoç et al.^30^ found that adding Al_2_O_3_ to the Al6061 matrix FGCMs improved wear resistance by 85.5%, particularly in FGCMs with 8 μm Al_2_O_3_ particles. In a study by Ardıçoğlu et al.,^31^ AA5083 matrix FGCMs with different step thicknesses and AA5083 foam materials were evaluated, revealing that the sample with a 2 mm step thickness had the highest hardness value, at 167 HB. Nagarjuna et al.^32^ investigated the effect of TiO_2_ addition on CrFeCuMnNi high-entropy alloys and reported that the incorporation of TiO_2_ ceramic enhanced the strength of the alloy. Similarly, Balaji et al.^33^ examined a hybrid nanoparticle composite produced by combining an aluminum alloy matrix with TiO_2_ and graphite nanoparticles. Their findings revealed that the hybrid nanocomposite exhibited superior mechanical and physical properties compared with the unreinforced aluminum alloy. Majzoobi et al.^34^ fabricated copper matrix composites containing micro- and nanosized TiO_2_ particles at different volume fractions (0, 2.5, and 5%) using the powder metallurgy method. They concluded that factors such as plastic strain, strain rate, fracture strain, reinforcement particle volume fraction, and particle aspect ratio significantly influenced damage formation in the material. Wen et al.^35^ studied WC- and B_4_C-reinforced copper matrix composites and determined that optimal thermal, mechanical, and tribological properties were achieved when the WC/B_4_C ratio was 5:3. In a related study, Wen et al.^36^ produced TiO_2_ and B_4_C-reinforced copper matrix composites by powder metallurgy and observed that the mechanical properties initially improved but then deteriorated as the TiB_2_/B_4_C ratio increased, with the best performance recorded at a ratio of 5:3. Liu et al.^37^ investigated the porosity that may occur in functionally graded materials (FGMs) by conducting a finite element analysis on functionally graded graphene-reinforced aluminum matrix composites. Naik et al.^38^ fabricated and compared two different types of ZrB_2_–B_4_C–SiC–LaB_6_ composites—layered FGMs and homogeneous NGCs—designed to withstand temperatures above 2000 °C. They reported that the FGM exhibited approximately 20% higher flexural strength than the NGC. Hamamcı et al.^39^ observed that in ten-layer iron-based FGMs produced via the powder metallurgy method, increasing the functional gradient accelerated thermal cracking and reduced mechanical strength. Erdemir et al.^40^ achieved a flexural strength of 1400 MPa in a two-layer Al2024/SiC FGM, where the upper layer contained 40% SiC reinforcement. Using the powder metallurgy method, Übeyli et al.^41^ found that SiC-reinforced FGM samples up to 25 mm thick failed to withstand bullet impact during ballistic tests and exhibited porosity arising from the production process.

In this study, the AA6082 alloy was selected as the matrix material due to its excellent weldability, high corrosion resistance, medium mechanical strength, and ability to improve mechanical properties when reinforced with other materials. B_4_C and TiO_2_ were selected as reinforcement materials, as well as TiO_2_ + B_4_C as hybrid reinforcement materials. These ceramics were chosen for their radiation shielding properties, which have been highlighted in previous studies,^42–46^ to investigate their effect on the mechanical properties of FGCMs/FGHCMs. In this study, the combinations of TiO_2_ and B_4_C ceramic reinforcement materials used at weight fractions of 0–50% have not been previously investigated in the literature. In addition to B_4_C and TiO_2_ ceramics, a uniquely selected B_4_C + TiO_2_ hybrid reinforcement was combined with the AA6082 alloy matrix at specific ratios. The distinctive aspect of this study lies in the evaluation of the mechanical and microstructural properties of functionally graded composite materials (FGCM) and functionally graded hybrid composite materials (FGHCM), produced by incorporating the selected reinforcement materials both individually and in combination at various weight ratios. Thus, this research aims to address the existing gap in the literature and contribute to the understanding of the properties of novel functionally graded material combinations. The objective of this work was to conduct a mechanical test (TRS), along with characterization analyses (SEM (fracture surface-microstructure) and EDS) on TiO_2_ and B_4_C-reinforced AA6082 functional-grade composite/hybrid materials produced by the powder metallurgy method.

## 2 Materials and Methods

### 2.1 Materials

The results obtained from the particle size analysis of the powders used in the production of TiO_2_ and B_4_C-reinforced AA6082 functional graded composite/hybrid materials are given in the study conducted by Özkan et al.^44^ Figures 1–3 show the SEM images of the AA6082, B_4_C, and TiO_2_ powders used in this study. The AA6082 matrix powder (Figure 1) exhibits a morphology that is nearly spherical. In contrast, the B_4_C ceramic powder (Figure 2) has an irregular morphology with sharp corners. The TiO_2_ ceramic powders (Figure 3) are spherical in shape. Due to the different particle size distributions of the powders, the particle sizes observed in the SEM images also differ accordingly.

**Figure 1:**
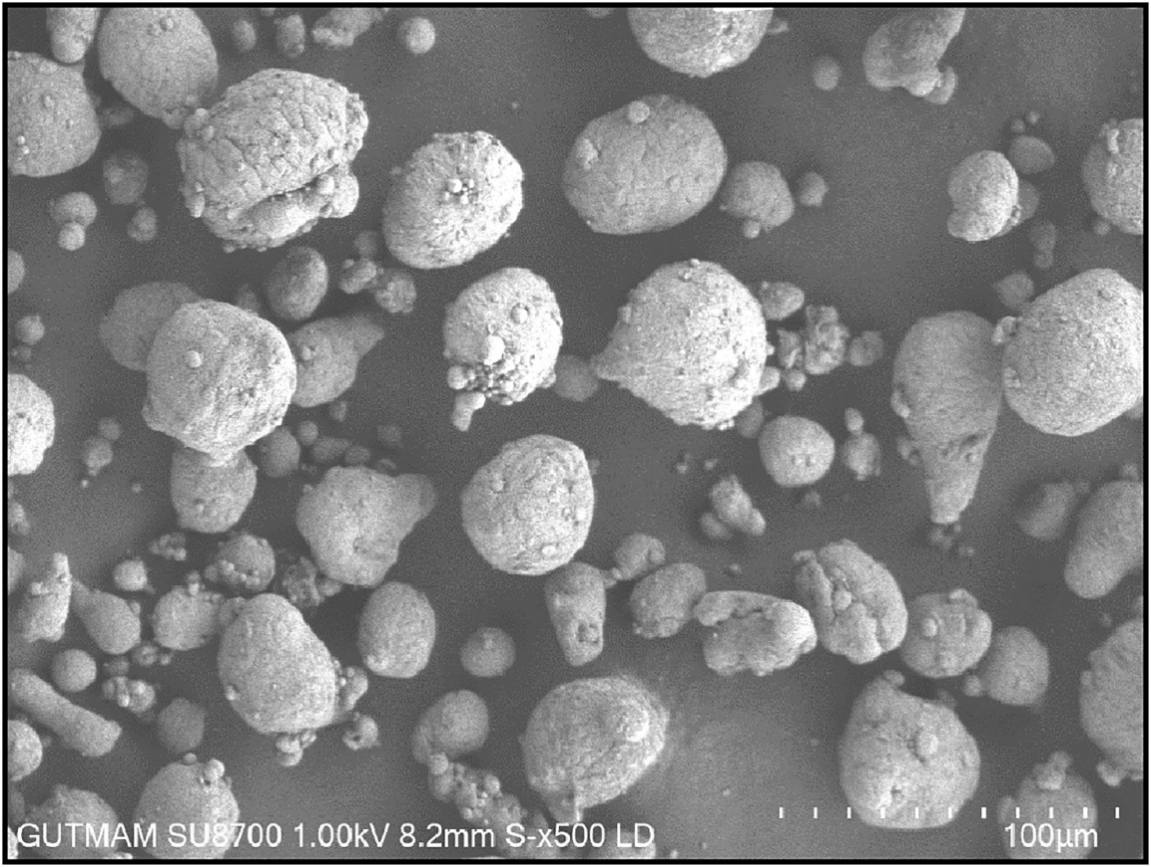
SEM image of AA6082 at ×500 magnification.

**Figure 2:**
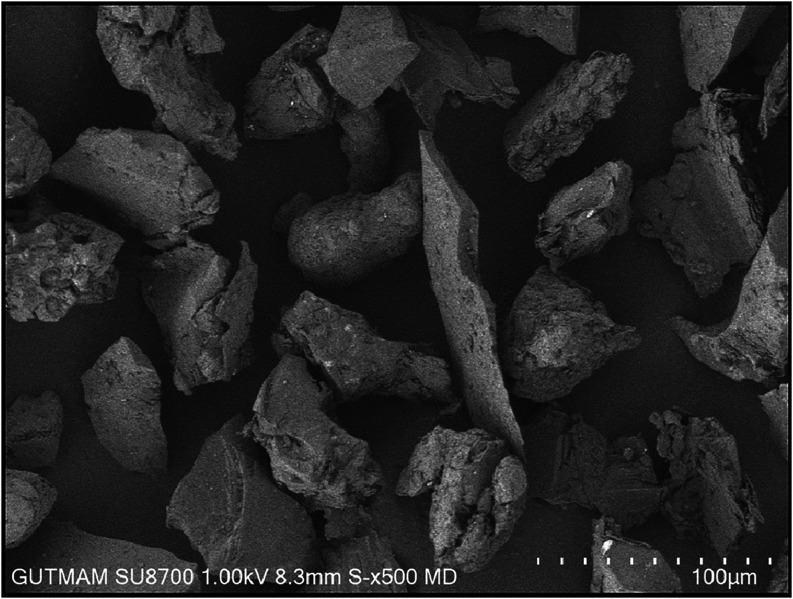
SEM image of B_4_C at ×500 magnification.

**Figure 3:**
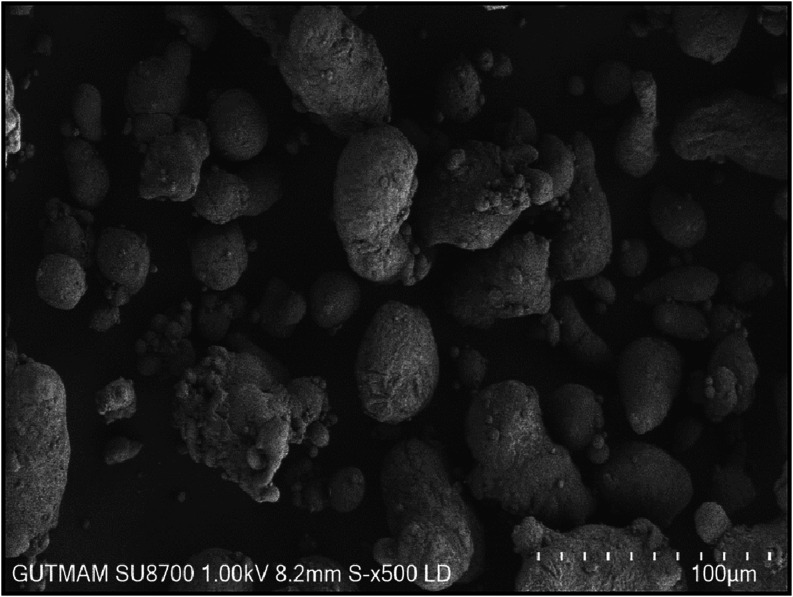
SEM image of TiO_2_ at ×500 magnification.

### 2.2 Method

Following the preparation of composite/hybrid mixture powders comprising AA6082, B_4_C, TiO_2_, and B_4_C + TiO_2_ (H), the hot pressing process employed a specially designed hydraulic press with a pressing capacity of 160 tons. The production studies of the samples were started at 450 MPa and 350 °C.^42–44^ The pressure and temperature were changed because the hot press used for sample production studies lost a lot of heat, the grains did not spread out, and the mold got damaged at high pressures. Production still happened, though. We tried to produce samples at a temperature of 580–730 °C, a pressure of 120–160 MPa, and for a duration of 1–3 h. We produced the final samples by selecting the optimum pressure and temperature. The final samples consist of six layers, each with gradually changing reinforcement ratios, and the AA6082 matrix is located in the lower layer (Figure 4).

1 $$relavite\;density\%=\frac{theoretical\;density}{Archimedes\;density}\times 100$$

2 %porosity=100%−relavitydensity%

**Figure 4:**
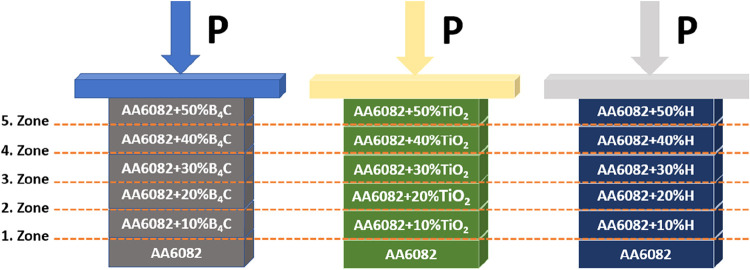
TiO_2_, B_4_C, and TiO_2_ + B_4_C reinforced FGCMs/FGHCM with an AA6082 matrix.

The experimental densities of the produced samples were calculated using the Archimedes’ principle and compared with the theoretical density. The relative density values and pore percentages of the produced materials were calculated using eqs 1 and 2, respectively.

## 3 Experimental Results and Discussion

### 3.1 XRD

Figure 5 shows the XRD graphs of the powders used in the study. It is understood that the peaks of the Al element in AA6082 occur in the lattice planes of (111), (200), (220), (311), and (222).^47^ It is understood from the XRD analysis data that there is a precipitate of Mg_2_Si as well as the Al element at the peak of AA6082 at 38.3°. The presence of Mg_5_Si_6_ precipitates was detected in the peak between 42 and 43°. The presence of Mg_2_Si and Mg_5_Si_6_ precipitates was detected at the 44.5° peak. 44–45° indicates the presence of the β-Al_2_Mg phase. TiO_2_ 2θ in the rutile phase has strong diffraction peaks at about 27, 36, and 55°, as shown by XRD patterns.^48^ When the XRD peaks of B_4_C ceramic powder analyzed at 10–90° (2θ) are examined, it is understood that the analyzed powder is B_4_C.^49^ B_4_C ceramics have 3 main peaks (∼23.4°–∼34.9°–∼37.7°).

**Figure 5:**
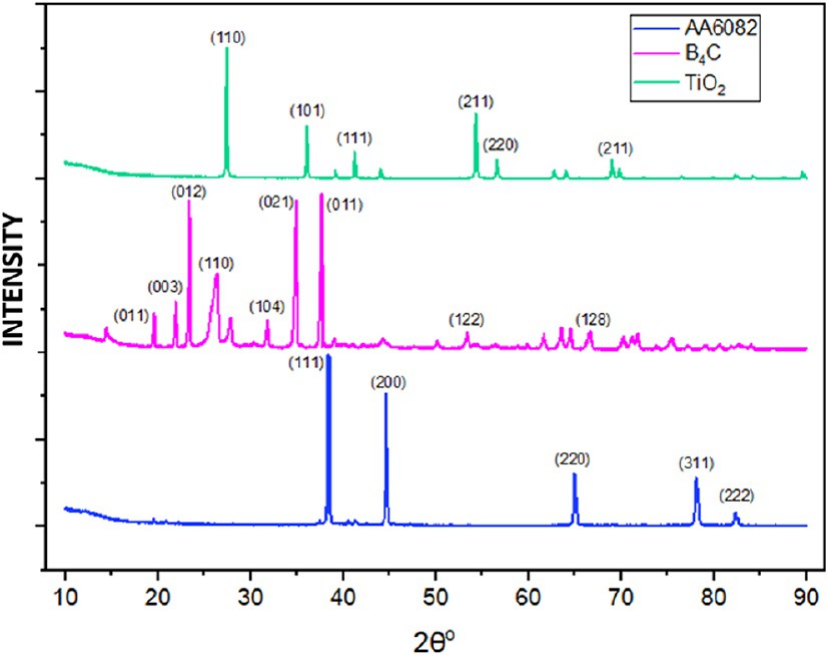
AA6082, B_4_C, and TiO_2_ XRD analysis.

### 3.2 Density

Among the samples produced,^46^ the one with the highest relative density of 99.68% is AA6082, without reinforcement material. Decreases were also detected in the relative density values calculated by considering the experimental density values and theoretical density values of FGHCM/FGCMs produced by adding reinforcement elements to AA6082 (Figure 6). The AA6082+ (0–50% wt.) B_4_C FGCM exhibited lower relative density values than the AA6082 material, primarily due to the increased proportion of ceramic particles resulting from the increased layer count. Due to the high hardness of the B_4_C ceramic material, it is very difficult to obtain a nonporous composite material through compression. The hard structure of the ceramic powder, with its varying B_4_C ratios (0 to 50% weight) in the AA6082 matrix material, is believed to negatively impact the compressibility of the powder metal blocks, increasing the friction force in the powder mixture and on the mold surfaces. When the graph is examined, it is understood that the most pores are in AA6082+ (0–50% wt.) TiO_2_ FGM. One of the main reasons for this is that the addition of hard particles such as TiO_2_ causes pores during production. Another reason is that the Dv(50) value of the TiO_2_ powders used is below 1 μm, and the surface energy of small-sized powder particles is higher; therefore, they are not effectively compacted during pressing. As the reinforcement ratio increased from the first layer (0% wt. TiO_2_) to the sixth layer (50% wt. TiO_2_), partial agglomerations formed during the mixing of the TiO_2_ ceramic with the matrix phase. This situation caused the formation of pores among the particles, the matrix material, and the reinforcement material. AA6082+ (0–50%) B_4_C + TiO_2_ FGHCM contains materials with 2 different mechanical properties, grain sizes, and melting temperatures. The relative density value of FGHCM is ∼96.63%, while the porosity value is ∼3.37%.

**Figure 6:**
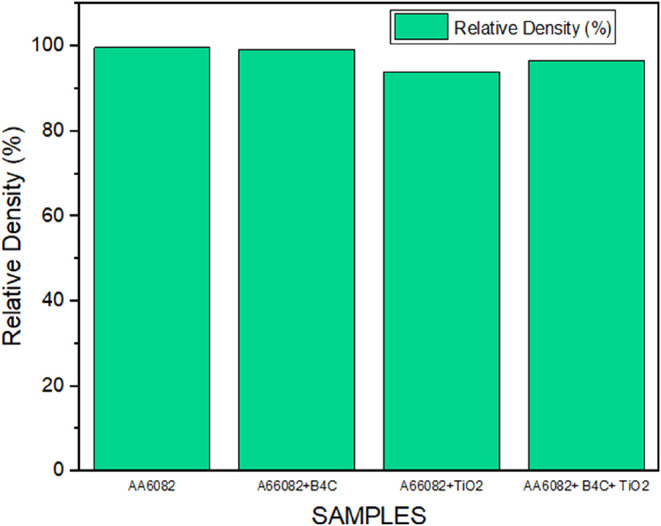
Comparison of the Archimedean density and the theoretical density.

The increase in the density in composite materials produced by incorporating high-density ceramic reinforcements into low-density matrix materials does not always result in enhanced mechanical strength; in some cases, it may even lead to a reduction in strength. This phenomenon is primarily associated with insufficient encapsulation of the reinforcement phases by the matrix and the formation of weak interfacial bonding. Such a weak interface between the reinforcement and the matrix adversely affects the load transfer mechanism, causing a nonuniform stress distribution within the material. Consequently, improper load transfer gives rise to local stress concentrations and premature crack initiation. As a result, the overall structural strength falls below expectations, and brittle fracture behavior becomes more pronounced with increasing reinforcement content. This phenomenon is illustrated in the fracture surface images presented in Figures 9–12.

### 3.3 TRS Test

Materials produced according to ASTM B528–05 standards were positioned as shown in Figure 7 and tested in the TRS device.

**Figure 7:**
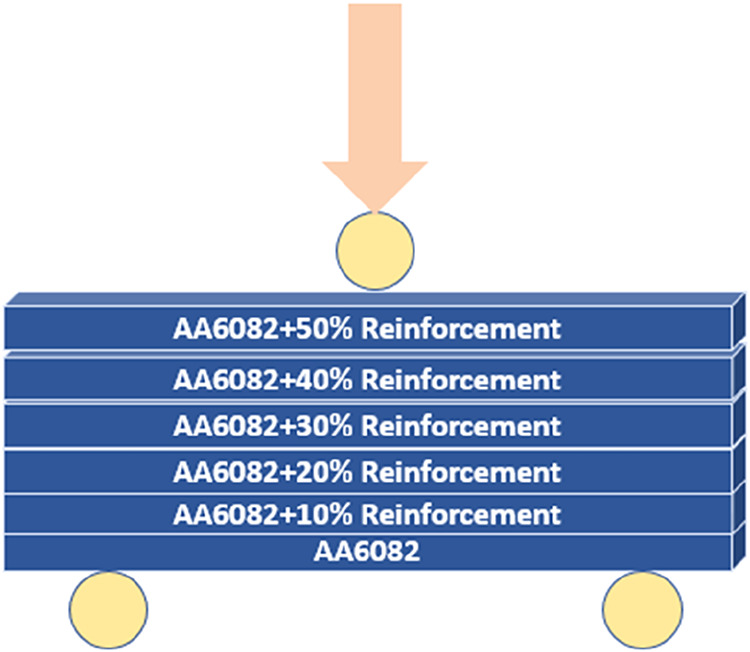
TRS analysis direction.

Experimental studies were carried out by applying a load from the sixth layer to the FGHCM/FGCM samples of each group produced in the study. Tests were repeated 3 times for each sample. The TRS and their averages obtained as a result of the experiment are listed in Figure 8.Among the obtained TRS values, the highest TRS was recorded for AA6082 at approximately 536.3 MPa, while the lowest value, around 366.9 MPa, was observed for the AA6082 + (0–50% wt %) B_4_C + TiO_2_-reinforced FGHCM/FGCMs material.

**Figure 8:**
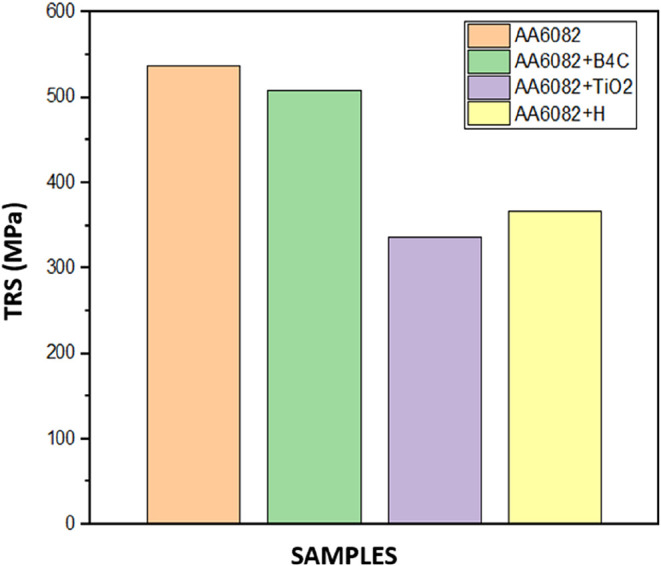
Results of the TRS.

As a result of the addition of reinforcement materials to the AA6082 matrix, the TRS decreased in all FGHCM/FGCMs. The reason for this is thought to be that the applied load is not transferred to the hard reinforcement materials (B_4_C and TiO_2_) added to the AA6082 ductile material. As a result of the matrix material not enveloping the reinforcement material, the applied load was not transmitted to the ceramic materials as it should have been. This caused the added reinforcement to create a notch effect within the material. The least decrease in TRS occurred in B_4_C-reinforced FGCM. This is due to the high hardness and strength of the B_4_C material, as well as the fact that the AA6082+ (0–50% wt.) FGCM contains fewer pores than the other samples. The greatest decrease in TRS occurred in the AA6082+ (0–50% wt.) TiO_2_ FGCM. The very high surface energy of TiO_2_ material, the high agglomeration of powder particles, and the notch effect of TiO_2_ ceramic on AA6082 matrix material are believed to be the main causes of this. The TRS of AA6082+ (0–50% wt.) B_4_C FGCM has ∼33.9% more TRS than AA6082+ (0–50% wt.) TiO_2_ material, Figure 9.

**Figure 9:**
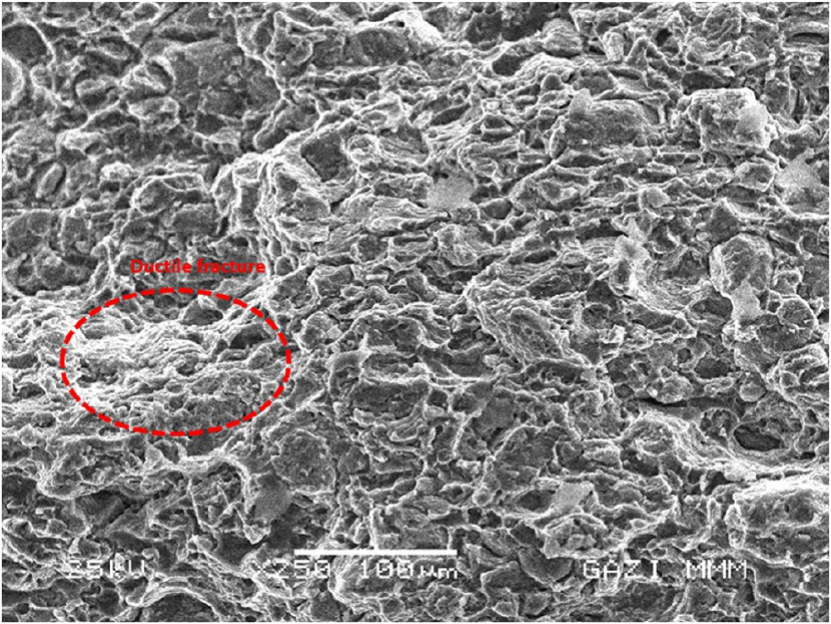
Fracture surface view of AA6082 at ×250 magnification.

### 3.4 Fracture Surface

Fractured surface SEM images of AA6082 matrix material and FGMs refracted from 6 layers, respectively, at 250× magnification are given in Figures 10–12.

**Figure 10:**
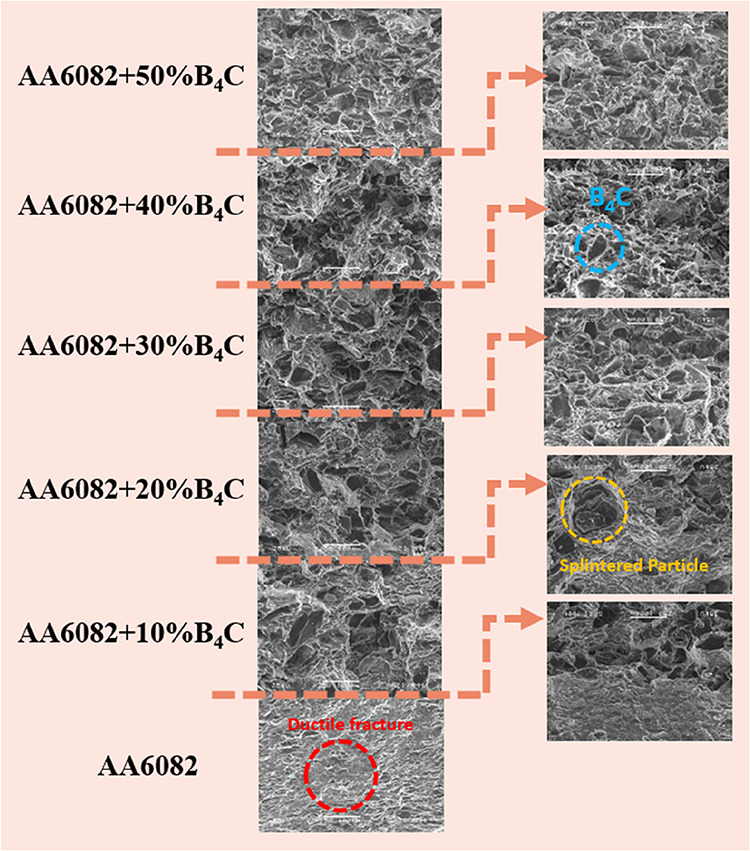
Fracture surface image of AA6082+ (0–50% wt.) B_4_C at ×250 magnification.

As expected from the AA6082 material (Figure 10), ductile fracture occurred during cross fracture. The reticular structure appearing in white indicates that the material is ductile. When Figure 10 is examined, the material in the AA6082 layer shows more ductile fracture behavior than the other layers. It is seen that the ductile behavior of the material increases in each layer with an increase of B_4_C, depending on the decreasing layers. This confirms the decrease in the flexural strength of the material with B_4_C reinforcement.

Figure 11 illustrates the ductility-broken state of the first layer, AA6082. It is seen that due to increasing TiO_2_ reinforcement ratios, the AA6082 matrix material breaks off from the material, and the TiO_2_ ceramic particles remain agglomerated. The ductile fracture behavior in the transition zones between layers 1–2 and 2–3 is seen more clearly than in the other interlayer regions.

**Figure 11:**
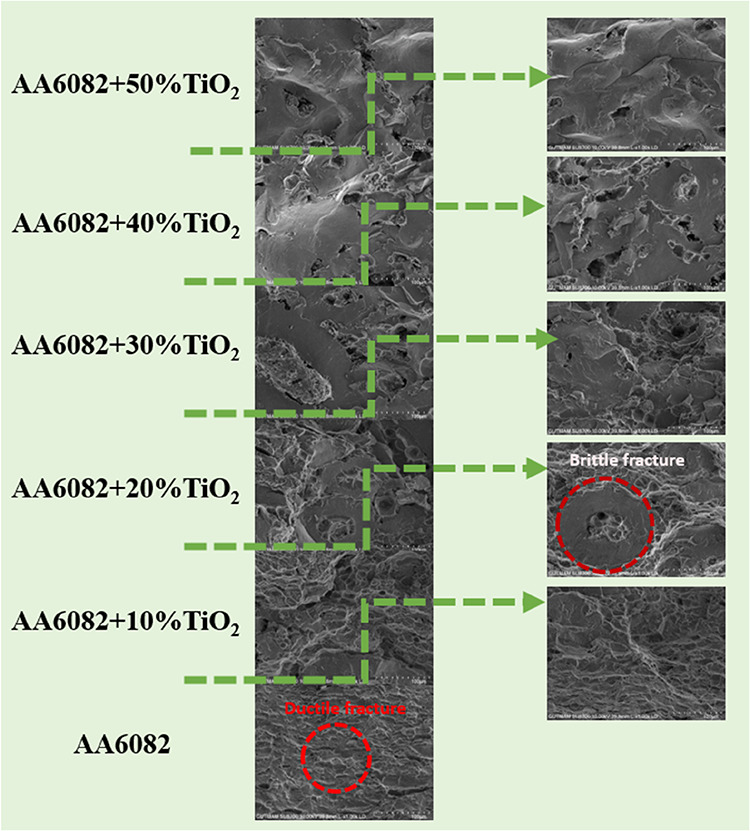
Fracture surface image of AA6082+ (0–50% wt.) TiO_2_ at ×250 magnification.

Examining Figure 12, we find that the material in the AA6082 layer exhibits more brittle fracture behavior than the other layers. We observe that the ductile behavior of the material increases in each layer as the B_4_C + TiO_2_ content increases, based on the increasing layers. It is seen that the layers show more ductile behavior in the transition regions than do the other layers. In the cracked surface images, it is seen that cracks occur in B_4_C ceramic, but it does not cause any effect on crack propagation. Due to the very small grain size of TiO_2_, the effect on the material could not be determined clearly. However, it has been determined that the B_4_C and TiO_2_ reinforcements cause a notch effect on the material and decrease the TRS.

**Figure 12:**
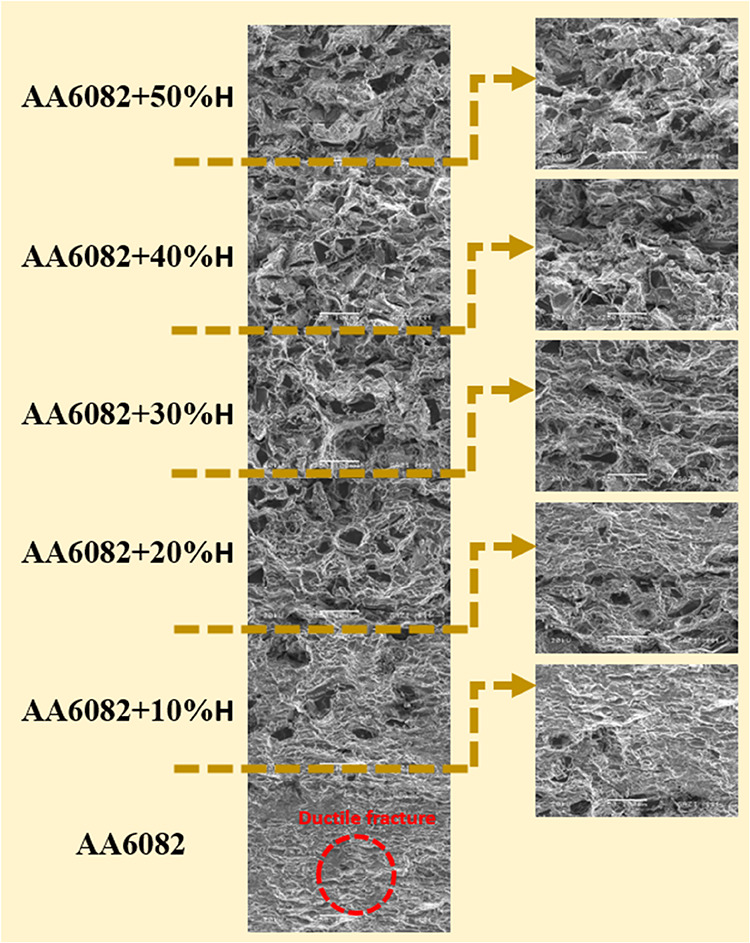
Fracture surface image of AA6082+ (0–50% wt.) H at ×250 magnification.

### 3.5 Hardness

In the previous study by Özkan et al.,^46^ the hardness values were taken as Vickers hardness.

### 3.6 EDS

Figure 13 provides the EDS mapping of the AA6082 material. The EDS analysis reveals that Mg is the primary alloying element in the region with its concentration in the grain glaze.

**Figure 13:**
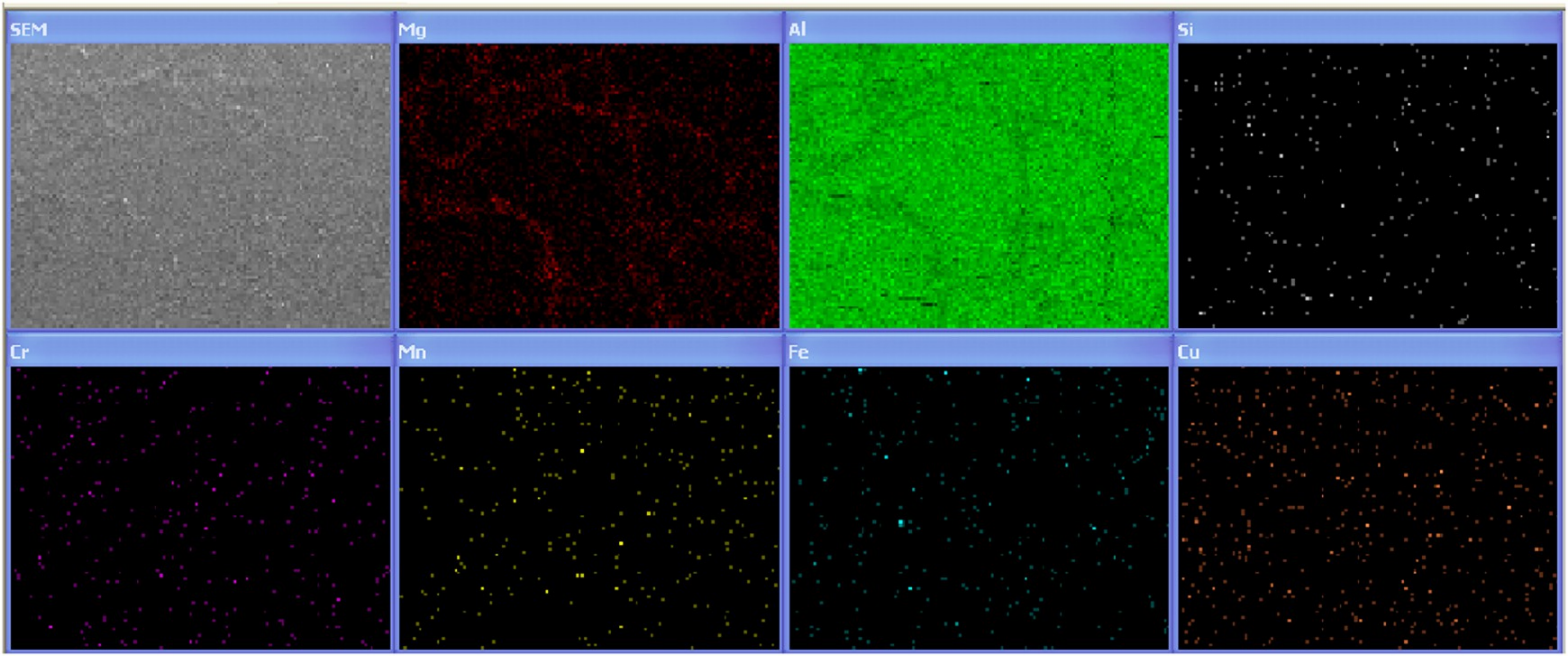
Elemental Map of AA6082.

In the previous study conducted by Özkan et al., the EDS analysis of the AA6082 matrix material, whose SEM image was taken, is given in Figure 14. EDS analysis from 4 different points was taken from the material surface at ×1000 magnification.

**Figure 14:**
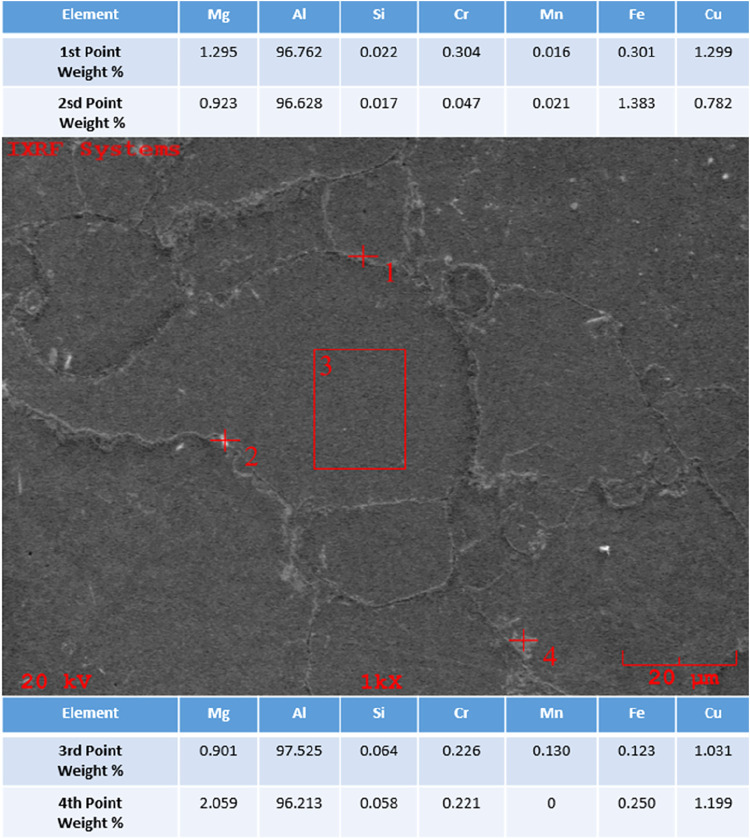
EDS image of AA6082 at ×1000 magnification.

Figure 15 shows the data obtained from EDS analysis of 4 different points at 1000× magnification of the second layer of AA6082+ (0–50% wt.) B_4_C, whose SEM images were taken by Özkan et al.^46^ It is clearly shown that the powder particle given at the first point is B_4_C.

**Figure 15:**
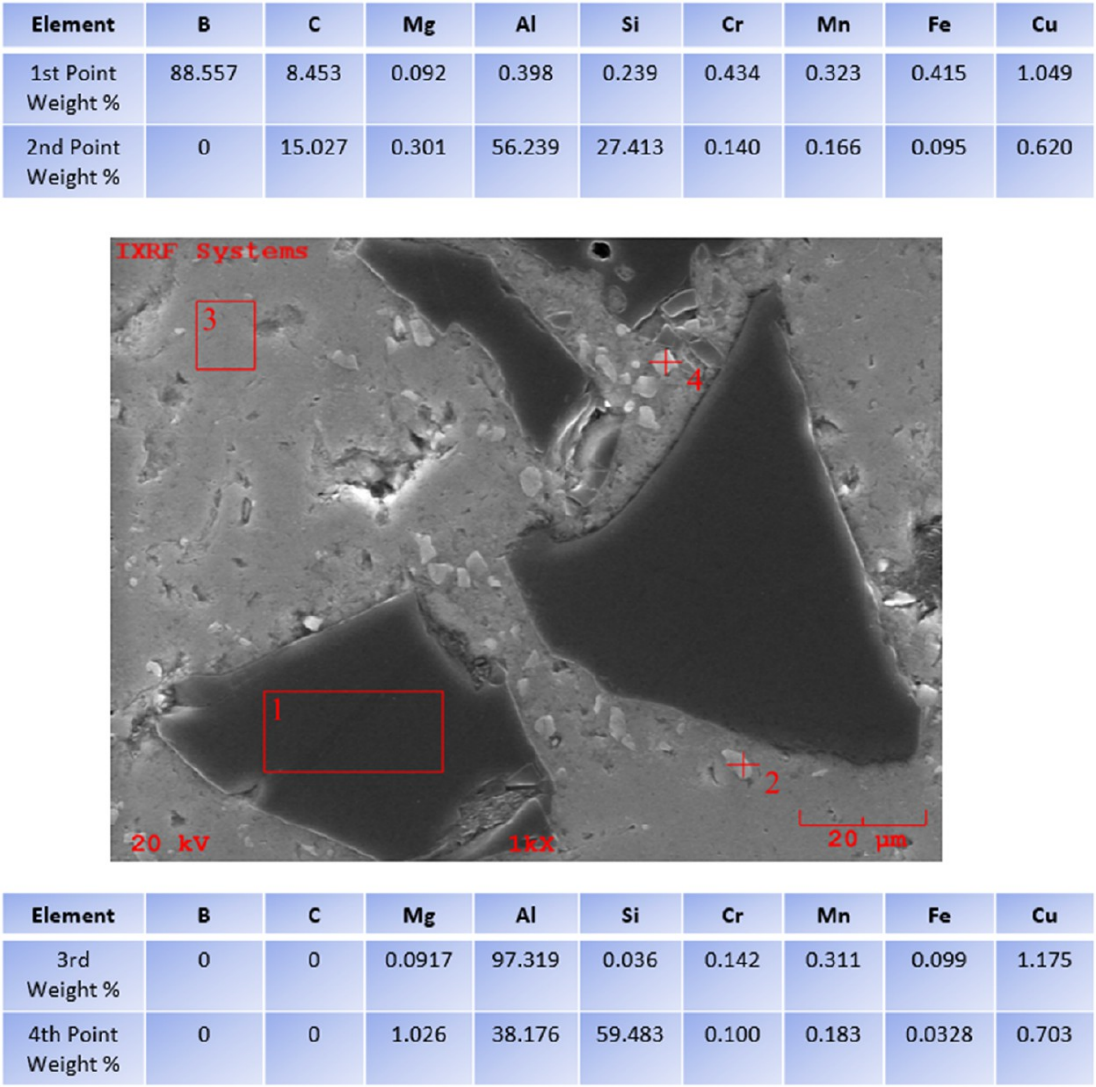
EDS image of AA6082+(0–50% weight) B_4_C at ×1000 magnification.

Figure 16 presents the data obtained from EDS analysis of 4 different points at 1000× magnification of the second layer of AA6082+ (0–50% wt.) TiO_2_ FGCM, whose SEM images were taken by Özkan et al.^42^ It is clearly seen that the powder particle given at the first point is an AA6082 matrix. It is understood from the EDS ratios of the leaf-shaped particle given at the second point that it is TiO_2_. When the ratios at the third and fourth points are examined, it is understood that TiO_2_ is clustered on AA6082.

**Figure 16:**
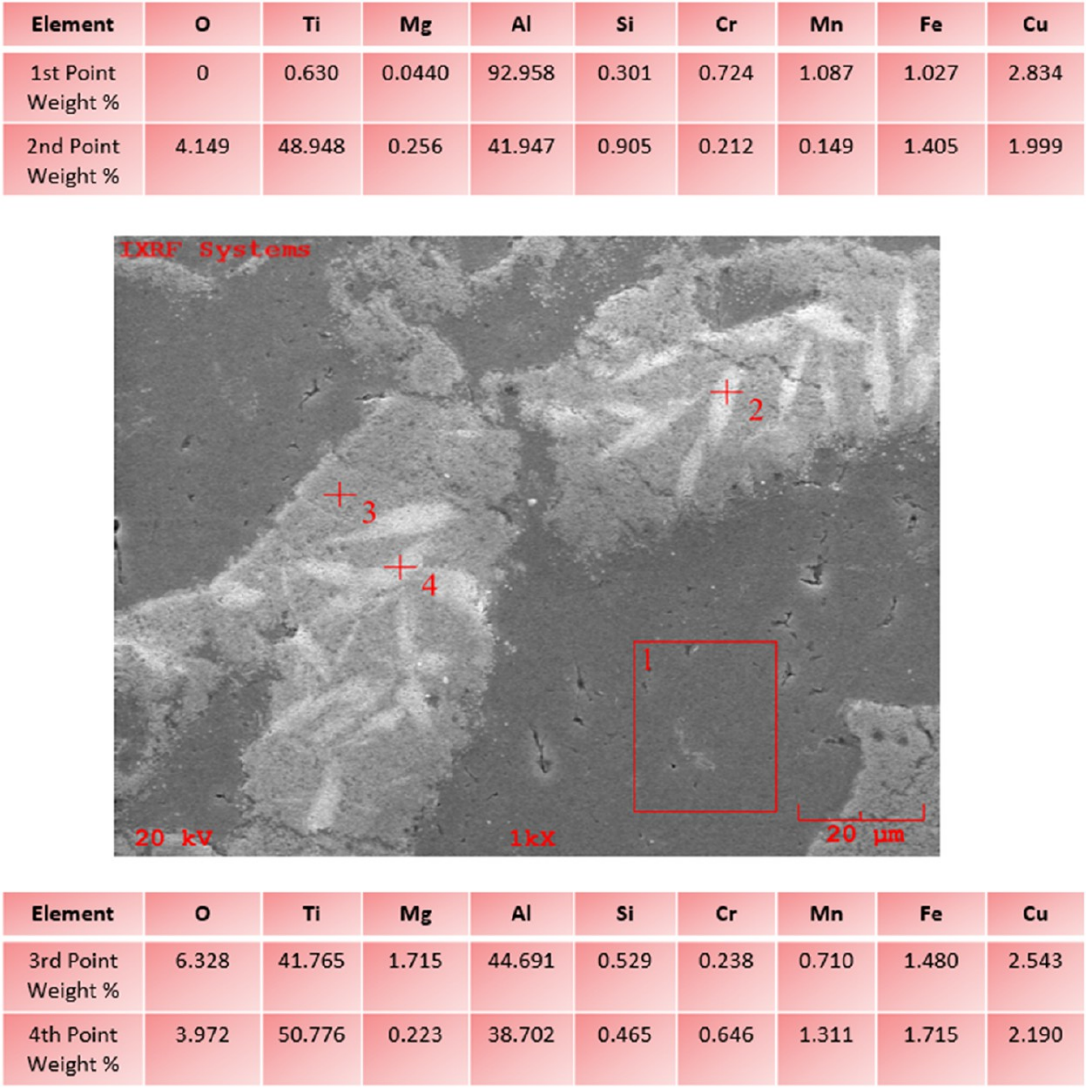
EDS image of AA6082+(0–50% weight) TiO_2_ at ×1000 magnification.

Figure 17 shows the data obtained by performing EDS analysis from 4 different points at 1000× magnification of the second layer of AA6082+ (0–50% wt.) H functionally graded hybrid composite material, whose SEM images were taken by Özkan et al.^46^ It is understood from the EDS analysis data that the powder particle given at the first point is a B_4_C ceramic and that there is a TiO_2_ powder particle on it. Ti and B elements were detected at the third point, but the reinforcing elements TiO_2_ and B_4_C could not be detected in the C and O elements of the ceramics. It is understood that at point 4, there is an AA6082 matrix material and trace amounts of the TiO_2_ ceramic.

**Figure 17:**
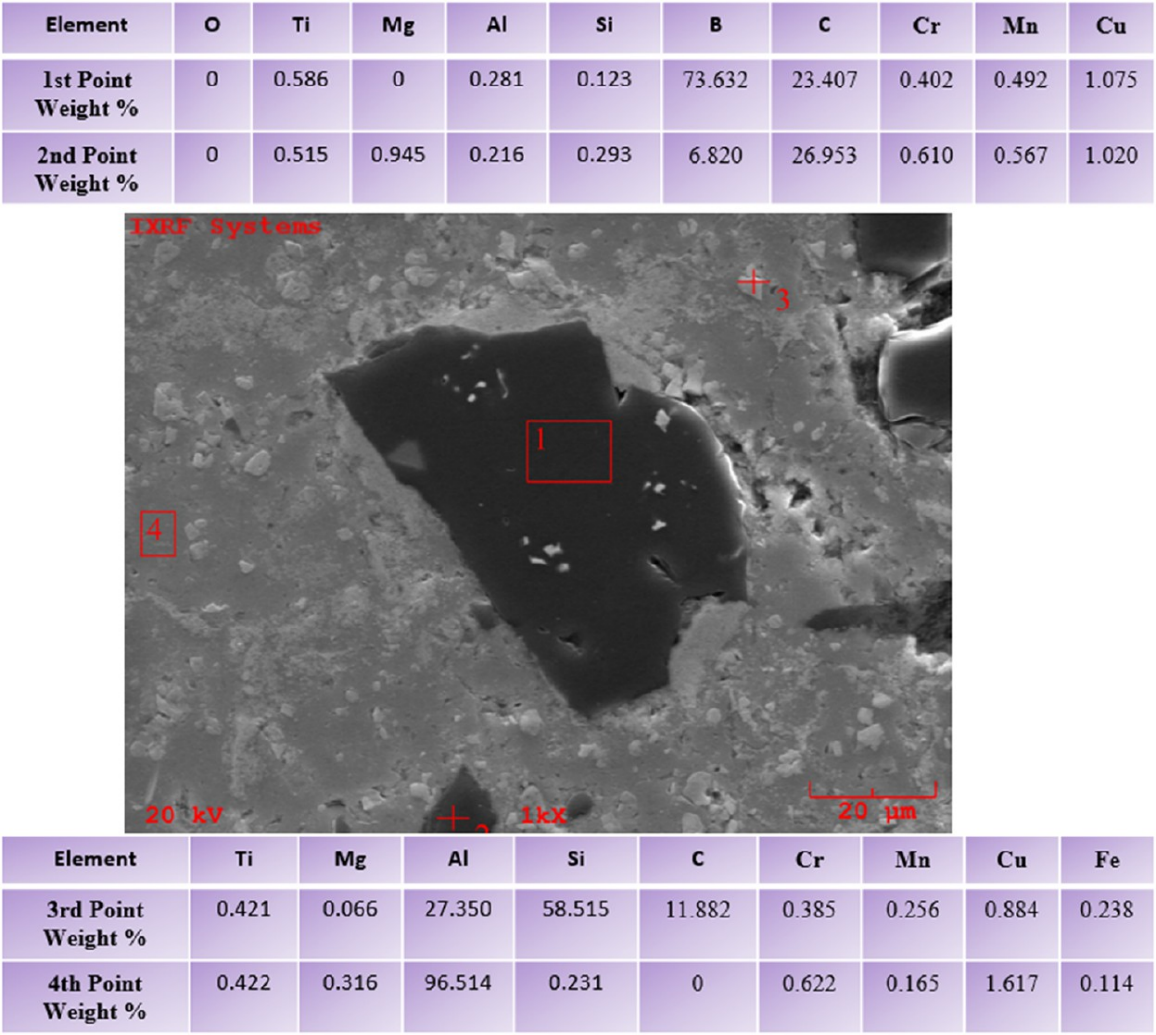
EDS image of AA6082+ (0–50% weight) H at ×1000 magnification.

## 4 Conclusion

In this study, AA6082, AA6082+ (0–50% wt.) B_4_C FGCM, AA6082+ (0–50% wt.) TiO_2_ FGCM, and AA6082+ (0–50% wt.) TiO_2_ + B_4_C FGHCM was successfully produced using the powder metallurgy method.

- Among the samples, AA6082 exhibited the highest relative density at 99.68%. The addition of reinforcement materials, such as B_4_C and TiO_2_, led to a decrease in the relative density of the composites, which can be attributed to the dispersion of the ceramic particles within the aluminum matrix and the resulting changes in microstructural characteristics.
- The fracture surface analysis revealed that the “notch effect” caused by the reinforcement materials, particularly B_4_C and TiO_2_, significantly influenced the fracture behavior of the composite. This effect arises due to the sharp edges and irregular shapes of the reinforcement particles, which create stress concentration points at the matrix-reinforcement interface. These localized stress points act as initiation sites for cracks under applied loads, leading to a brittle fracture behavior in regions with high reinforcement content.
- Modifying the particle size or shape could mitigate the notch effect and improve the fracture behavior. For instance, using finer particles with a more uniform size distribution could reduce stress concentrations by enabling better bonding and load transfer at the interface. Additionally, spherical or rounded particles could lower the severity of the notch effect by minimizing sharp edges, further enhancing the matrix-reinforcement interaction and delaying crack propagation. Adopting such modifications in reinforcement morphology could improve the composite’s TRS and overall toughness, making it more resistant to mechanical failure.
- The TRS decreased due to the increased porosity and agglomeration of ceramic particles, particularly in the TiO_2_-reinforced layers. This dual effect highlights the need for optimized particle distribution to balance TRS. Agglomeration and porosity remain key challenges, particularly in TiO_2_-reinforced composites. Addressing these challenges in future work could further optimize the TRS.
- The results suggest that while the ceramic reinforcements also introduce challenges in terms of load-bearing capacity, they do not uniformly carry the applied stresses. This is a common issue in composites with uneven phase distribution, leading to localized stress concentrations that weaken the overall material performance.
- Overall, the study highlights the potential of TiO_2_ and B_4_C-reinforced AA6082 composites for a range of engineering applications, particularly those requiring enhanced, tailored mechanical properties. However, for better performance, particularly in terms of load-bearing capacity, additional work is needed to refine the reinforcement distribution and optimize the fabrication processes.
- This study presents novel findings that fill a gap in the existing literature, demonstrating that the mechanical strength of functionally graded composite materials may be lower than that of the ductile matrix when the added reinforcement is not properly wetted by the matrix. • The results emphasize that the interaction at the matrix–reinforcement interface is a critical parameter in the design of FGCMs, underscoring the importance of improving interface compatibility through surface modification, additional processing techniques, and careful selection of suitable matrix materials.
